# On Topological Indices of Certain Families of Nanostar Dendrimers

**DOI:** 10.3390/molecules21070821

**Published:** 2016-06-24

**Authors:** Mohamad Nazri Husin, Roslan Hasni, Nabeel Ezzulddin Arif, Muhammad Imran

**Affiliations:** 1 School of Informatics and Applied Mathematics, University Malaysia Terengganu, Kuala Terengganu, Terengganu 21030, Malaysia; naz_reyhusin@yahoo.com; 2 Department of Mathematics, College of Computer Sciences and Mathematics, Tikrit University, Tikrit 34001, Iraq; nabarif@yahoo.com; 3 Department of Mathematics, School of Natural Sciences, National University of Sciences and Technology, Sector H-12, Islamabad 44000, Pakistan; imrandhab@gmail.com

**Keywords:** atom-bond connectivity index, geometric arithmetic index, dendrimer

## Abstract

A topological index of graph G is a numerical parameter related to G which characterizes its molecular topology and is usually graph invariant. In the field of quantitative structure-activity (QSAR)/quantitative structure-activity structure-property (QSPR) research, theoretical properties of the chemical compounds and their molecular topological indices such as the Randić connectivity index, atom-bond connectivity (ABC) index and geometric-arithmetic (GA) index are used to predict the bioactivity of different chemical compounds. A dendrimer is an artificially manufactured or synthesized molecule built up from the branched units called monomers. In this paper, the fourth version of ABC index and the fifth version of GA index of certain families of nanostar dendrimers are investigated. We derive the analytical closed formulas for these families of nanostar dendrimers. The obtained results can be of use in molecular data mining, particularly in researching the uniqueness of tested (hyper-branched) molecular graphs.

## 1. Introduction and Preliminary Results

There is a lot of mathematics involved in electrical and electronic engineering. Moreover, this depends on what area of electrical and electronic engineering: for example, there is a lot of abstract mathematics in communication theory, signal processing, networking and others. Networks involve nodes communicating with each other. A number of computers linked together form a network. Cell phone users form a network. Networking involves the study of the best way of implementing a network. Chemists are now equipped with a number of useful tools because of the merging branch of graph theory called chemical graph theory, e.g., molecular topological descriptors, indices and molecular topological polynomials. Many chemical structures and chemical compounds are usually modeled by a molecular graph to analyze underlying theoretical properties. A molecular graph is a pictorial diagram of the structural formula of a chemical structure in terms of graph theory, where the vertices represent the atoms of the given chemical compound and the edges represent the chemical bonds between the atoms. Cheminformatics is new subject which is a combination of chemistry, mathematics and information science. It studies Quantitative structure-activity (QSAR) and structure-property (QSPR) relationships that are used to predict the biological activities and properties of different chemical compounds. In the field of QSAR/QSPR research, theoretical properties of the chemical compounds and their molecular topological indices such as the Wiener index, Szaged index, Randić index, Zagreb index and ABC index are used to predict bioactivity of the chemical compounds. For more details about how to use the topological indices in predicating the bioactivity of chemical compounds, we refer the readers to consult [1,2]. For further details of the utilization of the topological indices and better understanding of the topic of research, the readers are hereby referred to the [3,4,5] for references on polymers.

A graph can be recognized by a numeric number, a polynomial, a sequence of number or a matrix. A topological index is a numeric number associated to a graph which completely describes the molecular topology of graph and this quantity is invariant under the automorphism of graphs. Among the interesting and most studied classes of topological indices in chemical graph theory are topological indices, which use the concept of distance called distance-based topological indices. The topological indices defined on the grounds of vertex degrees in a graph are called degree-based topological indices and the topological indices defined on the bases of counting are called counting related topological indices of graphs. Among these classes of topological indices described above, the degree-based topological indices have many more correlations to the chemical properties. In other words, a molecular topological index Top(G) of a graph is the number with the property that for each given graph H which is isomorphic to the graph G, we have Top(H) = Top(G). The concept of topological indices came from Wiener [6] when he was studying the boiling point of a member of alkane family, called paraffin. He named this topological index the path number. With the increase of research in chemical graph theory, the path number was given the name Wiener index later on. The Wiener index is the most investigated molecular topological index in chemical graph theory because of its interesting theoretical properties and wide range of applications, and because it is equal to the sum of the graph-theoretic distances between every pair of vertices in a graph G, see for details [7,8].

Dendrimers are among the most complex chemical and interesting structures and hyper-branched macromolecules, with a precise tailored architecture. Dendrimers have gained a wide range of application in supra-molecular chemistry, particularly in host guest reactions and the self-assembly process. Their application in chemistry, biology and nano-science are unlimited. Recently, the topological indices of certain families of dendrimers have been investigated in [9,10,11,12,13,14,15].

In this article, G is considered to be a simple and connected graph with vertex set V(G) and edge set E(G), d(u) is the degree of vertex u∈V(G) and $S_{u}=\sum_{v\in N_{G}\left(u\right)}d\left(v\right)$ where $N_{G}\left(u\right)=\{v\in V\left(G\right)|uv\in E\left(G\right)\}$. All notations in this paper are standard and mainly taken from books [8,16].

The very first and oldest degree based topological index is *Randić index* denoted by χ(G) introduced by Milan Randić in 1975 [17]. It is defined as

(1) $$\chi \left(G\right)=\underset{uv\in E\left(G\right)}{\sum }\frac{1}{\sqrt{d\left(u\right)d\left(v\right)}}$$

One of the well-known degree based topological indices is atom-bond connectivity (ABC) index introduced by Estrada et al. in [18], which was defined for modeling the enthalpy of formation of alkanes and was defined as follows:

(2) $$ABC\left(G\right)=\underset{uv\in E\left(G\right)}{\sum }\sqrt{\frac{d\left(u\right)+d\left(v\right)-2}{d\left(u\right)d\left(v\right)}}$$

Another well-known connectivity topological descriptor is the geometric-arithmetic (GA) index, which was introduced by Vukičević et al. in [19]. It was shown that its predictive power is somewhat better than the Randić index for many physicochemical properties like boiling point, entropy, enthalpy of vaporization, standard enthalpy of vaporization, enthalpy of formation and acentric factor, and it was defined as follows:

(3) $$GA\left(G\right)=\underset{uv\in E\left(G\right)}{\sum }\frac{2\sqrt{d\left(u\right)d\left(v\right)}}{d\left(u\right)+d\left(v\right)}$$

The fourth version of ABC index is introduced by Ghorbani et al. [20] and defined as:

(4) $$ABC_{4}\left(G\right)=\underset{uv\in E\left(G\right)}{\sum }\sqrt{\frac{S_{u}+S_{v}-2}{S_{u}S_{v}}}$$

Recently fifth version of GA index is proposed by Graovac et al. [21] and defined as:

(5) $$GA_{5}\left(G\right)=\underset{uv\in E\left(G\right)}{\sum }\frac{2\sqrt{S_{u}S_{v}}}{S_{u}+S_{v}}$$

Thus, the goal of this paper is to structurally characterize some dendrimers by investigations into these topological indices. The obtained results can be of use in molecular data mining, particularly in researching the uniqueness of tested (hyper-branched) molecular graphs.

Recently, there has been a huge amount of research activity about the ABC and GA topological indices, and their variants, for example, see [22,23]. The structure-sensitivity of degree-based molecular topological indices has been discussed in detail in [22]. $ABC_{4}$ and $GA_{5}$ indices for some families of nanostar dendrimers and polyphenylene dendrimers are discussed in [20,21,24]. For a detailed description, their properties and bounds of the molecular topological indices of various classes of graph, see [24,25,26,27,28,29,30,31,32]. In this paper, we give an explicit formula of the $ABC_{4}$ and $GA_{5}$ indices for certain nanostar dendrimers, namely PAMAM, tetrathiafulvalene and POPAM dendrimers.

## 2. Results and Discussion

**Figure 1:**
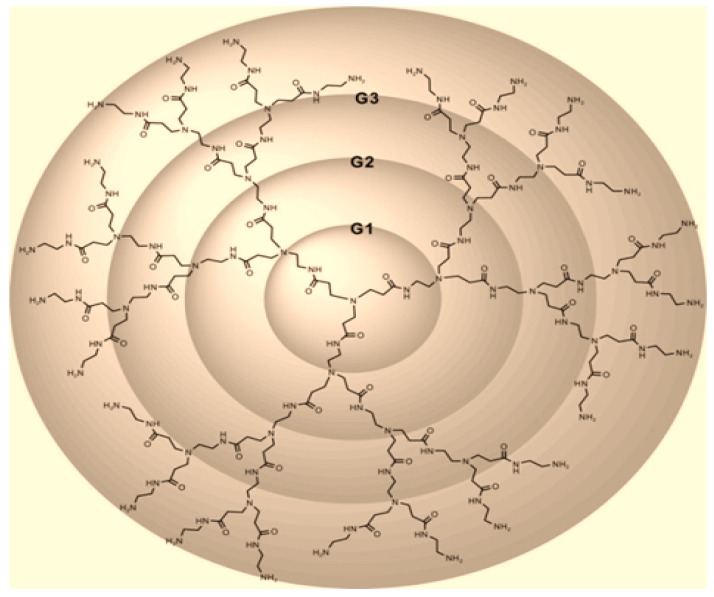
PAMAM dendrimer of generations $G_{n}$ with growth stages, $PD_{1}\left[3\right]$.

In this section, we study the $ABC_{4}$ and $GA_{5}$ indices of some families of dendrimers. We first consider the PAMAM dendrimer of generation $G_{n}$ with n growth stages, denoted by $PD_{1}\left[n\right]$ where n≥0. The number of vertices and edges in $PD_{1}\left[n\right]$ are $12\times 2^{n+2}-23$ and $12\times 2^{n+2}-24$ (see [13]). Denote an edge connecting a vertex i to a vertex j by (i,j)−edge, where $n_{i}$ denote the vertex i and $s_{ij}$ is the number of (i,j)−edges. The graph $PD_{1}\left[3\right]$ is shown in Figure 1.

In the following theorem, the exact formula of $ABC_{4}$ index for PAMAM dendrimer is computed.

**Theorem 1.**  Consider the PAMAM dendrimer $PD_{1}\left[n\right]$. The $ABC_{4}$ index of $PD_{1}\left[n\right]$ is equal to:

(6) $$ABC_{4}\left(PD_{1}\left[n\right]\right)=\left(\frac{87\sqrt{2}}{10}+\frac{\sqrt{15}}{2}+6\sqrt{\frac{2}{5}}+\frac{9}{2}\sqrt{\frac{7}{5}}+\frac{9\sqrt{30}}{10}\right)2^{n}-3\left(\sqrt{\frac{2}{5}}+\sqrt{\frac{7}{5}}+\frac{6\sqrt{2}}{5}+\frac{2\sqrt{30}}{10}\right)$$

**Proof.**  Let G be the graph of PAMAM dendrimer $PD_{1}\left[n\right]$. We have $\left|V\left(PD_{1}\left[n\right]\right)\right|=12\times 2^{n+2}-23$ and $\left|E\left(PD_{1}\left[n\right]\right)\right|=12\times 2^{n+2}-24$. We find the edge partition of the form (2,3), (3,4), (3,5), (4,5), (5,5), (5,6) for PAMAM dendrimer $PD_{1}\left[n\right]$ based on the degree sum of vertices lying at unit distance from end vertices of each edge. Table 1 explains such partition for $PD_{1}\left[n\right]$.

Now by using the partition given in Table 1, we can compute the $ABC_{4}\;$index for $PD_{1}\left[n\right]$. Since:

(7) $$ABC_{4}\left(G\right)=\underset{uv\in E\left(G\right)}{\sum }\sqrt{\frac{S_{u}+S_{v}-2}{S_{u}S_{v}}}$$

this implies that:

(8) $$\begin{array}{cc} ABC_{4}\left(PD_{1}\left[n\right]\right)= & \left(3\times 2^{n}\right)\sqrt{\frac{2+3-2}{2\times 3}}+\left(3\times 2^{n}\right)\sqrt{\frac{3+4-2}{3\times 4}}\\ & +\left(6\times 2^{n}-3\right)\sqrt{\frac{3+5-2}{3\times 5}}+\left(9\times 2^{n}-6\right)\sqrt{\frac{4+5-2}{4\times 5}}\\ & +\left(18\times 2^{n}-9\right)\sqrt{\frac{5+5-2}{5\times 5}}+\left(9\times 2^{n}-6\right)\sqrt{\frac{5+6-2}{5\times 6}} \end{array}$$

After an easy simplification, we obtain:

(9) $$\begin{array}{cc} ABC_{4}\left(PD_{1}\left[n\right]\right)= & \left(\frac{87\sqrt{2}}{10}+\frac{\sqrt{15}}{2}+6\sqrt{\frac{2}{5}}+\frac{9}{2}\sqrt{\frac{7}{5}}+\frac{9\sqrt{30}}{10}\right)2^{n}\\ & -3\left(\sqrt{\frac{2}{5}}+\sqrt{\frac{7}{5}}+\frac{6\sqrt{2}}{5}+\frac{2}{10}\sqrt{30}\right) \end{array}$$

This completes the proof.

The following theorem computes the $GA_{5}$ index of PAMAM dendrimer $PD_{1}\left[n\right]$.

**Theorem 2.**  Consider the PAMAM dendrimer $PD_{1}\left[n\right]$. The $GA_{5}$ index of $PD_{1}\left[n\right]$ is equal to:

(10) $$\begin{array}{cc} GA_{5}\left(PD_{1}\left[n\right]\right)= & \left(\frac{6\sqrt{6}}{5}+\frac{12\sqrt{3}}{7}+\frac{3\sqrt{15}}{2}+4\sqrt{5}+18+\frac{18\sqrt{30}}{11}\right)2^{n}\\ & -\left(\frac{3\sqrt{15}}{4}+\frac{8\sqrt{5}}{3}+9+\frac{12\sqrt{30}}{11}\right) \end{array}$$

**Proof.**  Let G be the graph of the PAMAM dendrimer $PD_{1}\left[n\right]$. The edge partition of PAMAM dendrimer $PD_{1}\left[n\right]$ based on the degree sum of vertices lying at unit distance from end vertices of each edge is given in Table 1. Now we apply the formula of $GA_{5}$ index for $PD_{1}\left[n\right]$. Since:

(11) $$GA_{5}\left(G\right)=\underset{uv\in E\left(G\right)}{\sum }\frac{2\sqrt{S_{u}S_{v}}}{S_{u}+S_{v}}$$

this implies that:

(12) $$\begin{array}{cc} GA_{5}\left(PD_{1}\left[n\right]\right)= & \frac{2\sqrt{2\times 3}}{2+3}\left(3\times 2^{n}\right)+\frac{2\sqrt{3\times 4}}{3+4}\left(3\times 2^{n}\right)+\frac{2\sqrt{3\times 5}}{3+5}\left(6\times 2^{n}-3\right)\\ & +\frac{2\sqrt{4\times 5}}{4+5}\left(9\times 2^{n}-6\right)+\frac{2\sqrt{5\times 5}}{5+5}\left(18\times 2^{n}-9\right)+\frac{2\sqrt{5\times 6}}{5+6}\left(9\times 2^{n}-6\right) \end{array}$$

After an easy simplification, we get:

(13) $$\begin{array}{cc} GA_{5}\left(PD_{1}\left[n\right]\right)= & \left(\frac{6\sqrt{6}}{5}+\frac{12\sqrt{3}}{7}+\frac{3\sqrt{15}}{2}+4\sqrt{5}+18+\frac{18\sqrt{30}}{11}\right)2^{n}\\ & -\left(\frac{3\sqrt{15}}{4}+\frac{8\sqrt{5}}{3}+9+\frac{12\sqrt{30}}{11}\right) \end{array}$$

This completes the proof.

**Figure 2:**
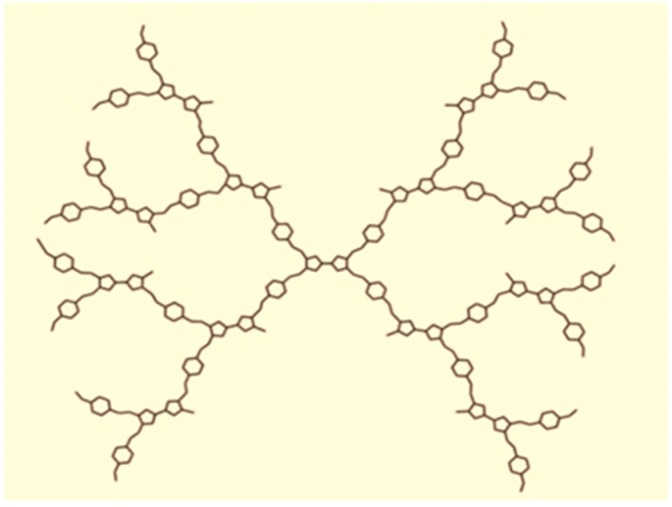
Tetrathiafulvalene dendrimer with 2-growth stages, $TD_{2}\left[2\right]$.

Now we shall compute the $ABC_{4}$ and $GA_{5}$ indices of tetrathiafulvalene dendrimer of generation $G_{n}$ with n growth stages, denoted by $TD_{2}\left[n\right]$ where n≥0. The graph of $TD_{2}\left[n\right]$ contains $31\times 2^{n+2}-74$ vertices and $35\times 2^{n+2}-85$ edges as shown in Figure 2 (see [15]). Table 2 shows the partition of edge set of tetrathiafulvalene dendrimer $TD_{2}\left[n\right]$ based on the degree sum of vertices lying at unit distance from end vertices of each edge, and by using this partition we compute the $ABC_{4}$ and $GA_{5}$ indices of tetrathiafulvalene dendrimer $TD_{2}\left[n\right]$.

In the following theorem, the exact formula of $ABC_{4}$ index for tetrathiafulvalene dendrimer $TD_{2}\left[n\right]$ is computed.

**Theorem 3.**  Consider tetrathiafulvalene dendrimer $TD_{2}\left[n\right]$. The $ABC_{4}$ index of $TD_{2}\left[n\right]$ is equal to

(14) $$\begin{array}{cc} ABC_{4}\left(TD_{2}\left[n\right]\right)= & \left(\frac{33\sqrt{2}}{10}+\frac{1}{3}\sqrt{\frac{7}{2}}+\frac{19\sqrt{3}}{21}+11\sqrt{\frac{3}{10}}+3\sqrt{\frac{2}{7}}+\frac{\sqrt{10}}{6}+8\sqrt{\frac{11}{42}}\right)2^{n+2}\\ & -\left(\frac{4}{3}\sqrt{\frac{7}{2}}+\frac{32\sqrt{2}}{5}+24\sqrt{\frac{3}{10}}+8\sqrt{\frac{2}{7}}+\frac{2\sqrt{10}}{3}+24\sqrt{\frac{11}{42}}+\frac{10\sqrt{3}}{7}\right) \end{array}$$

**Proof.**  Let G be the graph of tetrathiafulvalene dendrimer $TD_{2}\left[n\right]$. We have $\left|V\left(TD_{2}\left[n\right]\right)\right|=31\times 2^{n+2}-74$ and $\left|E\left(TD_{2}\left[n\right]\right)\right|=35\times 2^{n+2}-85$ . We find the edge partition of the form (2,4), (3,6), (4,6), (5,5), (5,6), (5,7), (6,6), (5,7), (7,7) for tetrathiafulvalene dendrimer $TD_{2}\left[n\right]$ based on the degree sum of vertices lying at unit distance from end vertices of each edge. Table 2 explains such partition for $TD_{2}\left[n\right]$.

Now by using the partition given in Table 2, we can compute the $ABC_{4}\;$index for $TD_{2}\left[n\right]$. Since

(15) $$ABC_{4}\left(G\right)=\underset{uv\in E\left(G\right)}{\sum }\sqrt{\frac{S_{u}+S_{v}-2}{S_{u}S_{v}}}$$

this implies that

(16) $$\begin{array}{cc} ABC_{4}\left(TD_{2}\left[n\right]\right)= & \left(2^{n+2}\right)\sqrt{\frac{2+4-2}{2\times 4}}+\left(2^{n+2}-4\right)\sqrt{\frac{3+6-2}{3\times 6}}+\left(2^{n+2}\right)\sqrt{\frac{4+6-2}{4\times 6}}\\ & +\left(7\times 2^{n+2}-16\right)\sqrt{\frac{5+5-2}{5\times 5}}+\left(11\times 2^{n+2}-24\right)\sqrt{\frac{5+6-2}{5\times 6}}\\ & +\left(3\times 2^{n+2}-8\right)\sqrt{\frac{5+7-2}{5\times 7}}+\left(2^{n+2}-4\right)\sqrt{\frac{6+6-2}{6\times 6}}\\ & +\left(8\times 2^{n+2}-24\right)\sqrt{\frac{6+7-2}{6\times 7}}+\left(2\times 2^{n+2}-5\right)\sqrt{\frac{7+7-2}{7\times 7}} \end{array}$$

After an easy simplification, we have

(17) $$\begin{array}{cc} ABC_{4}\left(TD_{2}\left[n\right]\right)= & \left(\frac{33\sqrt{2}}{10}+\frac{1}{3}\sqrt{\frac{7}{2}}+\frac{19\sqrt{3}}{21}+11\sqrt{\frac{3}{10}}+3\sqrt{\frac{2}{7}}+\frac{\sqrt{10}}{6}+8\sqrt{\frac{11}{42}}\right)2^{n+2}\\ & -\left(\frac{4}{3}\sqrt{\frac{7}{2}}+\frac{32\sqrt{2}}{5}+24\sqrt{\frac{3}{10}}+8\sqrt{\frac{2}{7}}+\frac{2\sqrt{10}}{3}+24\sqrt{\frac{11}{42}}+\frac{10\sqrt{3}}{7}\right) \end{array}$$

This completes the proof.

Following theorem computes the $GA_{5}$ index of tetrathiafulvalene dendrimer $TD_{2}\left[n\right]$.

**Theorem 4.**  Consider the tetrathiafulvalene dendrimer $TD_{2}\left[n\right]$. The $GA_{5}$ index of $PD_{1}\left[n\right]$ is equal to

(18) $$\begin{array}{cc} GA_{5}\left(TD_{2}\left[n\right]\right)= & \left(\frac{4\sqrt{2}}{3}+\frac{2\sqrt{6}}{5}+9+2\sqrt{30}+\frac{\sqrt{35}}{2}+\frac{16\sqrt{43}}{13}\right)2^{n+2}\\ & -\left(\frac{8\sqrt{2}}{3}+25+\frac{48\sqrt{30}}{11}+\frac{4\sqrt{35}}{3}+\frac{48\sqrt{43}}{13}\right) \end{array}$$

**Proof.**  Let G be the graph of tetrathiafulvalene dendrimer $TD_{2}\left[n\right]$. The edge partition of tetrathiafulvalene dendrimer $TD_{2}\left[n\right]$ based on the degree sum of vertices lying at unit distance from end vertices of each edge is given in Table 2. Now we apply the formula of $GA_{5}$ index for $TD_{2}\left[n\right]$. Since:

(19) $$GA_{5}\left(G\right)=\underset{uv\in E\left(G\right)}{\sum }\frac{2\sqrt{S_{u}S_{v}}}{S_{u}+S_{v}},$$

this implies that:

(20) $$\begin{array}{cc} GA_{5}\left(TD_{2}\left[n\right]\right)= & \frac{2\sqrt{2\times 4}}{2+4}\left(2^{n+2}\right)+\frac{2\sqrt{3\times 6}}{3+6}\left(2^{n+2}-4\right)+\frac{2\sqrt{4\times 6}}{4+6}\left(2^{n+2}\right)\\ & +\frac{2\sqrt{5\times 5}}{5+5}\left(7\times 2^{n+2}-16\right)+\frac{2\sqrt{5\times 6}}{5+6}\left(11\times 2^{n+2}-24\right)\\ & +\frac{2\sqrt{5\times 7}}{5+7}\left(3\times 2^{n+2}-8\right)+\frac{2\sqrt{6\times 6}}{6+6}\left(2^{n+2}-4\right)\\ & +\frac{2\sqrt{6\times 7}}{6+7}\left(8\times 2^{n+2}-24\right)+\frac{2\sqrt{7\times 7}}{7+7}\left(2\times 2^{n+2}-5\right) \end{array}$$

After an easy simplification, we have:

(21) $$\begin{array}{cc} GA_{5}\left(TD_{2}\left[n\right]\right)= & \left(\frac{4\sqrt{2}}{3}+\frac{2\sqrt{6}}{5}+9+2\sqrt{30}+\frac{\sqrt{35}}{2}+\frac{16\sqrt{43}}{13}\right)2^{n+2}\\ & -\left(\frac{8\sqrt{2}}{3}+25+\frac{48\sqrt{30}}{11}+\frac{4\sqrt{35}}{3}+\frac{48\sqrt{43}}{13}\right) \end{array}$$

This completes the proof.

**Figure 3:**
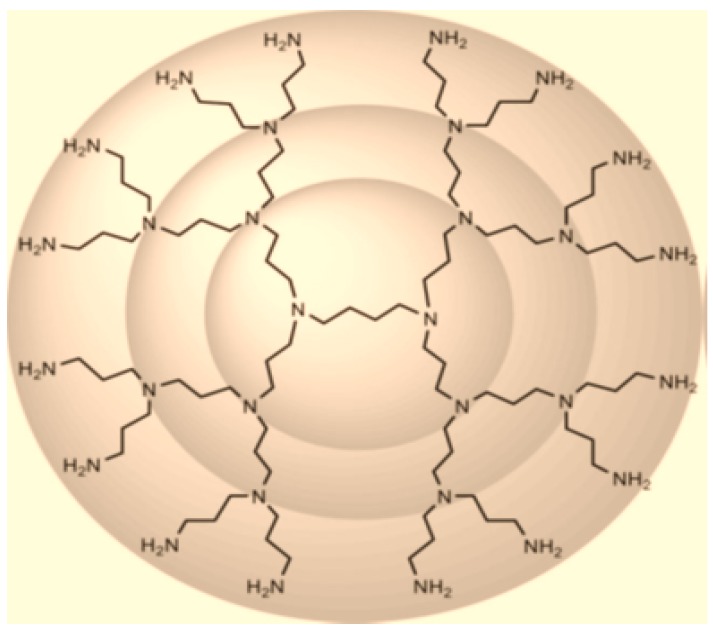
POPAM dendrimer of generations $G_{n}$ with two growth stages, $POD_{2}\left[2\right]$.

We shall now determine the $ABC_{4}$ and $GA_{5}$ indices of POPAM dendrimers, denoted by $POD_{2}\left[n\right]$ where n≥0. The number of vertices and edges in $POD_{2}\left[n\right]$ are $2^{n+5}-10$ and $2^{n+5}-11$, respectively (see [14]). Figure 3 shows the graph of POPAM dendrimer $POD_{2}\left[n\right]$ with 2 growth stages. Table 3 shows the partition of edge set of POPAM dendrimer $POD_{2}\left[n\right]$ based on the degree sum of vertices lying at unit distance from end vertices of each edge, and by using this partition we compute the $ABC_{4}$ and $GA_{5}$ indices of POPAM dendrimer $POD_{2}\left[n\right]$.

In the following theorem, the exact formula of $ABC_{4}$ index for POPAM dendrimer $POD_{2}\left[n\right]$ is computed.

**Theorem 5.**  Consider POPAM dendrimer $POD_{2}\left[n\right]$. The $ABC_{4}$ index of $POD_{2}\left[n\right]$ is equal to

(22) $$ABC_{4}\left(POD_{2}\left[n\right]\right)=\left(\frac{\sqrt{2}}{2}+\frac{\sqrt{15}}{6}+3\sqrt{\frac{3}{10}}\right)2^{n+2}+\left(\frac{\sqrt{6}}{4}+6\sqrt{\frac{7}{5}}\right)2^{n}-\left(6\sqrt{\frac{7}{5}}+6\sqrt{\frac{3}{10}}\right)$$

**Proof.**  Let G be the graph of POPAM dendrimer $POD_{2}\left[n\right]$. We have $\left|V\left(POD_{2}\left[n\right]\right)\right|=2^{n+5}-10$ and $\left|E\left(POD_{2}\left[n\right]\right)\right|=2^{n+5}-11$. We find the edge partition of the form (2,3), (3,4), (4,4), (4,5) and (5,6) for POPAM dendrimer $POD_{2}\left[n\right]$ based on the degree sum of vertices lying at unit distance from end vertices of each edge. Table 3 explains such partition for $POD_{2}\left[n\right]$.

Now, by using the partition given in Table 3, we can compute the $ABC_{4}\;$index for $POD_{2}\left[n\right]$. Since:

(23) $$ABC_{4}\left(G\right)=\underset{uv\in E\left(G\right)}{\sum }\sqrt{\frac{S_{u}+S_{v}-2}{S_{u}S_{v}}}$$

this implies that

(24) $$\begin{array}{cc} ABC_{4}\left(POD_{2}\left[n\right]\right)= & \left(2^{n+2}\right)\sqrt{\frac{2+3-2}{2\times 3}}+\left(2^{n+2}\right)\sqrt{\frac{3+4-2}{3\times 4}}+\left(1\right)\sqrt{\frac{4+4-2}{4\times 4}}\\ & +\left(3\times 2^{n+2}-6\right)\sqrt{\frac{4+5-2}{4\times 5}}+\left(3\times 2^{n+2}-6\right)\sqrt{\frac{5+6-2}{5\times 6}} \end{array}$$

After an easy simplification, we get

(25) $$ABC_{4}\left(POD_{2}\left[n\right]\right)=\left(\frac{\sqrt{2}}{2}+\frac{\sqrt{15}}{6}+3\sqrt{\frac{3}{10}}+\frac{3}{2}\sqrt{\frac{7}{5}}\right)2^{n+2}-\left(6\sqrt{\frac{7}{5}}+6\sqrt{\frac{3}{10}}-\frac{\sqrt{6}}{4}\right)$$

This completes the proof.

Following theorem computes the $GA_{5}$ index of POPAM dendrimers $POD_{2}\left[n\right]$.

**Theorem 6.**  Consider POPAM dendrimers $POD_{2}\left[n\right]$. The $GA_{5}$ index of $POD_{2}\left[n\right]$ is equal to:

(26) $$GA_{5}\left(POD_{2}\left[n\right]\right)=\left(\frac{2\sqrt{6}}{5}+\frac{4\sqrt{3}}{7}+\frac{4\sqrt{5}}{3}+\frac{6\sqrt{30}}{11}\right)2^{n+2}-\left(\frac{8\sqrt{5}}{3}+\frac{12\sqrt{30}}{11}-1\right)$$

**Proof.**  Let G be the graph of POPAM dendrimers $POD_{2}\left[n\right]$. The edge partition of POPAM dendrimers $POD_{2}\left[n\right]$ based on the degree sum of vertices lying at unit distance from end vertices of each edge is given in Table 3. Now we apply the formula of $GA_{5}$ index for $POD_{2}\left[n\right]$. Since:

(27) $$GA_{5}\left(G\right)=\underset{uv\in E\left(G\right)}{\sum }\frac{2\sqrt{S_{u}S_{v}}}{S_{u}+S_{v}}$$

This implies that:

(28) $$\begin{array}{cc} GA_{5}\left(POD_{2}\left[n\right]\right)= & \frac{2\sqrt{2\times 3}}{2+3}\left(2^{n+2}\right)+\frac{2\sqrt{3\times 4}}{3+4}\left(2^{n+2}\right)+\frac{2\sqrt{4\times 4}}{4+4}\left(1\right)\\ & +\frac{2\sqrt{4\times 5}}{4+5}\left(3\times 2^{n+2}-6\right)+\frac{2\sqrt{5\times 6}}{5+6}\left(3\times 2^{n+2}-6\right) \end{array}$$

After an easy simplification, we get:

(29) $$GA_{5}\left(POD_{2}\left[n\right]\right)=\left(\frac{2\sqrt{6}}{5}+\frac{4\sqrt{3}}{7}+\frac{4\sqrt{5}}{3}+\frac{6\sqrt{30}}{11}\right)2^{n+2}-\left(\frac{8\sqrt{5}}{3}+\frac{12\sqrt{30}}{11}-1\right)$$

This completes the proof.

## 3. Conclusions

Molecular topology (or topological indices) has widely demonstrated its high performance in the discovery and design of new drugs. With this paper we seek to contribute to a better knowledge of molecular topology among mathematicians. Moreover: molecular topology can be employed to look for drugs that heal, in principle, any disease, based on the structural information provided by known active compounds. Once more, ‘pure’ mathematics comes to the rescue in practical problems, this time in the form of graph theory. If the book of nature is written with numbers, then molecular topology is certainly one way of reading it. Particularly, in this paper, we have considered some families of dendrimers, namely, PAMAM, tetrathiafulvalene and POPAM dendrimers, and studied their topological indices. The analytical closed formulas of the fourth version of atom-bond connectivity index $ABC_{4}$ and the fifth version of geometric-arithmetic index $GA_{5}$ for these families of dendrimers are determined. The obtained results can be of useful in molecular data mining, particularly in researching the uniqueness of tested (hyper-branched) molecular graphs. In the future, we are interested to study and compute topological indices of various families of dendrimers/networks which will be quite useful in understanding their underlying topologies.

## Figures and Tables

**Table 1:** Edge partition of PAMAM dendrimer, $PD_{1}\left[n\right]$ based on degree sum of neighbors of end vertices of each edge.

$\left(S_{u},S_{v}\right)$ Where uv∈E(G)	Number of Edges
(2,3)	$3\times 2^{n}$
(3,4)	$3\times 2^{n}$
(3,5)	$6\times 2^{n}-3$
(4,5)	$9\times 2^{n}-6$
(5,5)	$18\times 2^{n}-9$
(5,6)	$9\times 2^{n}-6$

**Table 2:** Edge partition of tetrathiafulvalene dendrimer $TD_{2}\left[n\right]$ based on degree sum of neighbors of end vertices of each edge.

$\left(S_{u},S_{v}\right)$ Where uv∈E(G)	Number of Edges
(2,4)	$2^{n+2}$
(3,6)	$2^{n+2}-4$
(4,6)	$2^{n+2}$
(5,5)	$7\times 2^{n+2}-16$
(5,6)	$11\times 2^{n+2}-24$
(5,7)	$3\times 2^{n+2}-8$
(6,6)	$2^{n+2}-4$
(6,7)	$8\times 2^{n+2}-24$
(7,7)	$2\times 2^{n+2}-5$

**Table 3:** Edge partition of POPAM dendrimer, $POD_{2}\left[n\right]$ based on degree sum of neighbors of end vertices of each edge.

$\left(S_{u},S_{v}\right)$ Where uv∈E(G)	Number of Edges
(2,3)	$2^{n+2}$
(3,4)	$2^{n+2}$
(4,4)	1
(4,5)	$3\times 2^{n+2}-6$
(5,6)	$3\times 2^{n+2}-6$
